# Screening of the Key Genes and Signalling Pathways for Diabetic Nephropathy Using Bioinformatics Analysis

**DOI:** 10.3389/fendo.2022.864407

**Published:** 2022-07-12

**Authors:** Zukai Li, Junxia Feng, Jinting Zhong, Meizhi Lu, Xuejuan Gao, Yunfang Zhang

**Affiliations:** ^1^ The Third School of Clinical Medicine, Southern Medical University, Guangzhou, China; ^2^ Department of Nephrology, Affiliated Huadu Hospital, Southern Medical University (People’s Hospital of Huadu District), Guangzhou, China; ^3^ The Central Laboratory, Affiliated Huadu Hospital, Southern Medical University (People’s Hospital of Huadu District), Guangzhou, China; ^4^ Key Laboratory of Functional Protein Research of Guangdong Higher Education Institutes and Ministry of Education (MOE) Key Laboratory of Tumor Molecular Biology, Institute of Life and Health Engineering, Jinan University, Guangzhou, China

**Keywords:** diabetic nephropathy, bioinformatic analysis, differentially expressed genes, FN1, biomarkers

## Abstract

**Background:**

This study aimed to identify biological markers for diabetic nephropathy (DN) and explore their underlying mechanisms.

**Methods:**

Four datasets, GSE30528, GSE47183, GSE104948, and GSE96804, were downloaded from the Gene Expression Omnibus (GEO) database. The differentially expressed genes (DEGs) were identified using the “limma” package, and the “RobustRankAggreg” package was used to screen the overlapping DEGs. The hub genes were identified using cytoHubba of Cytoscape. Logistic regression analysis was used to further analyse the hub genes, followed by receiver operating characteristic (ROC) curve analysis to predict the diagnostic effectiveness of the hub genes. Correlation analysis and enrichment analysis of the hub genes were performed to identify the potential functions of the hub genes involved in DN.

**Results:**

In total, 55 DEGs, including 38 upregulated and 17 downregulated genes, were identified from the three datasets. Four hub genes (*FN1*, *CD44*, *C1QB*, and *C1QA*) were screened out by the “UpSetR” package, and *FN1* was identified as a key gene for DN by logistic regression analysis. Correlation analysis and enrichment analysis showed that *FN1* was positively correlated with four genes (*COL6A3*, *COL1A2*, *THBS2*, and *CD44*) and with the development of DN through the extracellular matrix (ECM)–receptor interaction pathway.

**Conclusions:**

We identified four candidate genes: *FN1*, *C1QA*, *C1QB*, and *CD44*. On further investigating the biological functions of *FN1*, we showed that *FN1* was positively correlated with *THBS2*, *COL1A2*, *COL6A3*, and *CD44* and involved in the development of DN through the ECM–receptor interaction pathway. *THBS2*, *COL1A2*, *COL6A3*, and *CD44* may be novel biomarkers and target therapeutic candidates for DN.

## Introduction

Diabetic nephropathy (DN) is one of the most important microvascular diseases in diabetes and has become the chief cause of the end-stage renal disease (1). The clinical incidence of DN is high, and its main features include proteinuria, increased serum creatinine (Scr), and decreased glomerular filtration rate (GFR) (2). Studies have revealed that the progression of DN is related to genetic factors as well as to hemodynamic and metabolic changes (3). At present, the primary diagnostic marker of DN is microalbuminuria (MA). However, there is still a controversy regarding whether the appearance of MA represents kidney damage and whether MA inevitably progresses to obvious proteinuria and chronic renal function decline. Therefore, it is necessary to identify other biological markers that can predict kidney damage.

Microarray technology and bioinformatics analysis have enabled the identification of genetic alterations at the genome level. In recent years, bioinformatics methods have been widely used to analyse microarray data to identify the differentially expressed genes (DEGs) in addition to various other analyses. In this study, we analysed multiple microarray datasets, including GSE30528, GSE47183, GSE104948, and GSE96804. The common DEGs were identified in GSE30528, GSE47183, and GSE104948 datasets. The hub genes were identified by protein–protein interaction (PPI) network analysis and ten algorithms of the cytoHubba plugin. The predictive capability of the biomarker was analysed by receiver operating characteristic (ROC) curve and logistic regression analyses. The correlation between the biomarker and other genes was analysed by Pearson’s rank correlation using R software. Furthermore, Gene Ontology (GO), Kyoto Encyclopedia of Genes and Genomes (KEGG), and Gene Set Enrichment Analysis (GSEA) were used to determine the potential functions of the biomarker.

## Materials and Methods

### Microarray Data and Preprocessing

Microarray data were downloaded from the Gene Expression Omnibus (GEO) database (http://www.ncbi.nlm.nih.gov/geo) (4): GSE30528 (Affymetrix GPL571 Platform-HG-U133A_2), GSE47183 (Affymetrix GPL14663 Platform-Affy_HGU133A_CDF_ENTREZG_10), GSE104948 (Affymetrix GPL22945 Platform-HG-U133_Plus_2), and GSE96804 (Affymetrix GPL17586 Platform-Affymetrix Human Transcriptome Array 2.0). The GSE30528 dataset consisted of nine glomerular tissue samples from DN patients and 13 glomerular tissue samples from control human kidneys. GSE47183 comprised seven glomerular tissue samples from DN patients and 14 glomerular tissue samples from patients who had undergone tumour nephrectomy. GSE104948 consisted of seven glomerular tissue samples from DN patients and three glomerular tissue samples from patients who had undergone tumour nephrectomy. Raw data of GSE30528, GSE47183, and GSE104948 datasets were downloaded and read with the “oligo” package, and the Robust Multi-array Average (RMA) algorithm was used for background correction and data normalisation. GSE96804 dataset contained 41 glomerular tissue samples from DN patients and 20 glomerular tissue samples from control human kidneys. Series matrix files of the GSE96804 were downloaded. Platform annotation file was used to convert the probe expression matrix into a gene expression matrix. The details of all data are shown in **Table 1**.

**Table 1:** The information of four datasets.

GEO	Platform	Tissue (*Homo sapiens*)	Samples (number)			Experiment type	Attribute	Author/reference
			Total	DN	Control			
GSE30528	GPL571	Glomerular	22	9	13	Array	Test	Woroniecka KI (5)
GSE47183	GPL14663	Glomerular	21	7	14	Array	Test	Ju W (6)
GSE104948	GPL22945	Glomerular	10	7	3	Array	Test	Grayson PC (7)
GSE96804	GPL17586	Glomerular	61	41	20	Array	Validation	Pan Y (8)

GEO, Gene Expression Omnibus.

### Identification of the Differentially Expressed Genes

“Limma” package was utilised to identify the DEGs between the glomerular tissues of DN patients and controls. A p-value <0.05 and |log FC (fold change)| > 1 were considered statistically significant. The “Pheatmap” package was used to construct a heatmap of the DEGs, and “ggplot2” package was used to establish a volcano plot of the DEGs. The RobustRankAggreg (RRA) method was used to integrate and analyse the three datasets (GSE30528, GSE47183, and GSE104948) to obtain the common DEGs. The upregulated and downregulated gene lists were sorted by logFC in each dataset. Subsequently, all gene lists were integrated by the RobustRankAggreg package.

### Screening of Hub Genes

The overlapping DEGs of GSE30528, GSE47183, and GSE104948 were analysed by a search tool for the retrieval of interacting genes/proteins (STRING) (https://www.string-db.org/) to predict the PPI network and to determine the possible relationships between them (confidence level 0.4). The cytoHubba plugin of Cytoscape (v 3.9.0) was used to score each node gene by 10 randomly selected algorithms, including MNC (Maximum Neighbourhood Component), Degree, MCC (Maximal Clique Centrality), EcCentricity, EPC (Edge Percolated Component), Closeness, BottleNeck, Betweenness, Radiality, and Stress. The top 15 hub genes from each algorithm were used to screen hub genes through the “UpSetR” package.

### Screening and Verification of the Biomarker

The gene expression matrices of GSE30528, GSE47183, and GSE104948 were combined to obtain a new Merge dataset. The merged data were preprocessed by “sva” package to remove batch effects (9). Afterwards, logistic regression analysis was performed on the hub genes using the “rms” package, and variables with significant differences were screened out as key genes. Thereafter, the predictive effect of the key gene was further validated in the dataset GSE96804.

### Correlation Analysis and Enrichment Analysis

To further explore the functions of the identified key gene, the association of the key gene with other genes was explored using Pearson’s rank correlation analysis. The top 50 genes with the strongest associations were selected for GO and KEGG pathway function enrichment analyses. P.adjust < 0.05 was considered significant enrichment. Moreover, GSEA was used to further determine the potential functions of the key gene involved in DN. For this process, the “clusterProfiler” package was used. The KEGG sets and reference sets were used for functional enrichment analysis.

## Results

### Differentially Expressed Gene Screening

**Figure 1:**
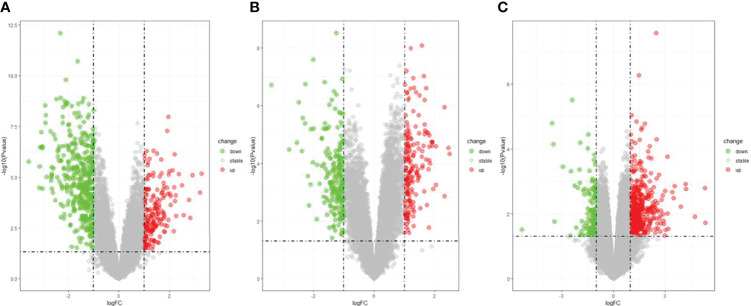
The volcano plot of DEGs with consistency from GSE30528 **(A)**, GSE47183 **(B)**, and GSE104948 **(C)**. DEGs, differentially expressed genes.

**Figure 2:**
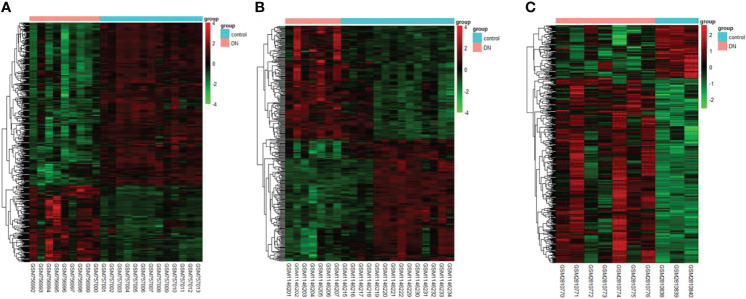
The heatmap of clustering analysis of DEGs with consistency from GSE30528 **(A)**, GSE47183 **(B)**, and GSE104948 **(C)**. DEGs, differentially expressed genes.

The DEGs were screened by “limma” package (p < 0.05 and |log FC| > 1).The GSE30528 dataset contained 554 DEGs, including 177 upregulated genes and 377 downregulated genes. The GSE47183 dataset contained 371 DEGs with 178 upregulated genes and 193 downregulated genes. The GSE104948 dataset contained 537 DEGs, including 417 upregulated genes and 120 downregulated genes. The DEGs of the three datasets are shown in **Figure 1**, and the heatmap of the DEGs is shown in **Figure 2**.

**Figure 3:**
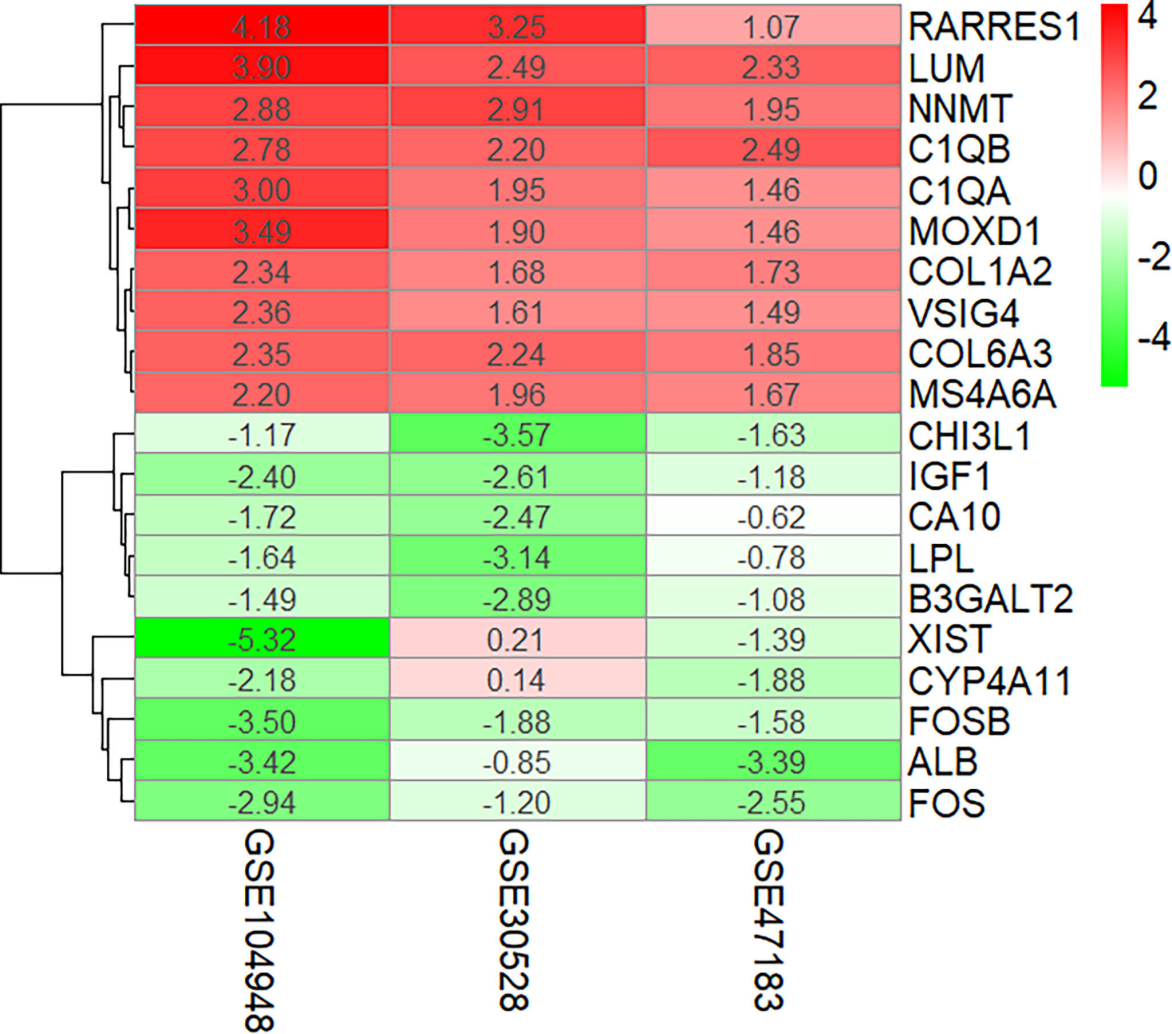
Ten upregulated and downregulated DEGs of the three datasets determined by “RRA.” DEGs, differentially expressed genes.

The overlapping DEGs were screened by the RRA method, which takes the intersection of multiple sequenced gene sets to screen out the genes that exhibit differences and rank high in each dataset. Finally, 55 integrated DEGs, comprising 38 upregulated genes and 17 downregulated genes, were identified by the RRA method as shown in **Table 2** and **Figure 3**.

**Table 2:** Fifty-five differentially expressed genes (DEGs) were identified from three datasets.

DEGs	Gene names
Upregulated	*LUM*, *NNMT*, *C1QB*, *RARRES1*, *COL6A3*, *C1QA*, *MOXD1*, *COL1A2*, *VSIG4*, *MS4A6A*, *ADH1B*, *TGFBI*, *FN1*, *C3*, *CPA3*, *COMP*, *CD163*, *MS4A4A*, *MUC1*, *DDX3Y*, *PID1*, *RGS4*, *TYROBP*, *IGFBP3*, *KRT19*, *TRAF3IP3*, *LTF*, *COL15A1*, *COL1A1*, *THBS2*, *CD44*, *CCR2*, *RNASE6*, *CRIP1*, *LYZ*, *RPS4Y1*, *WFDC2*, *IL10RA*
Downregulated	*ALB*, *FOS*, *CHI3L1*, *CYP4A11*, *FOSB*, *IGF1*, *LPL*, *XIST*, *CA10*, *B3GALT2*, *TNNT2*, *NPHS1*, *NDNF*, *ZNF595*, *HPD*, *HPGD*, *APOD*

### Protein–Protein Interaction Network Analysis and Hub Gene Screening

**Figure 4:**
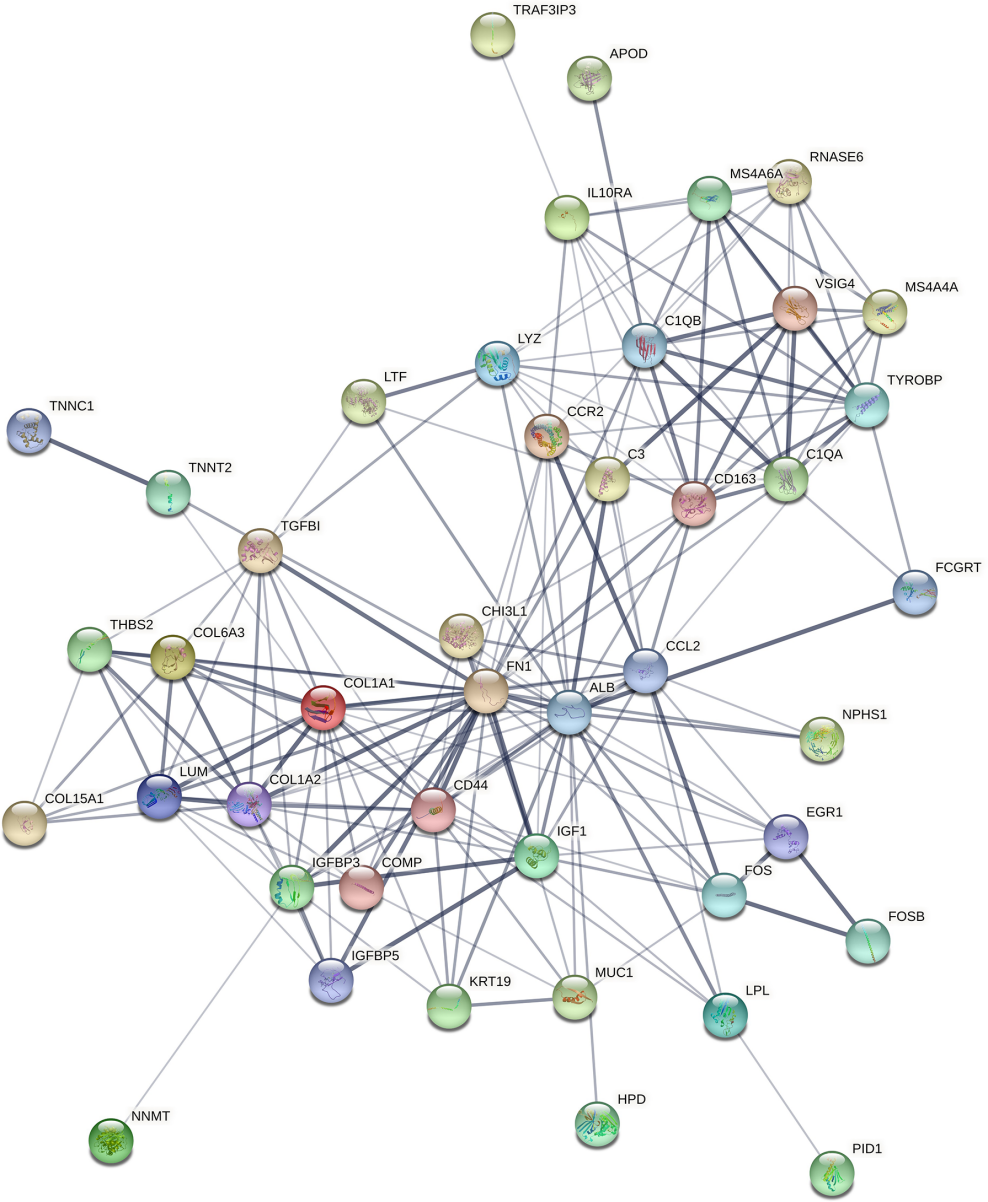
The PPI network of overlapping DEGs of three microarray datasets. Circles represent genes, lines represent interactions between gene-encoded proteins, and line thickness represents confidence in interactions between proteins. PPI, protein–protein interaction; DEGs, differentially expressed genes.

**Figure 5:**
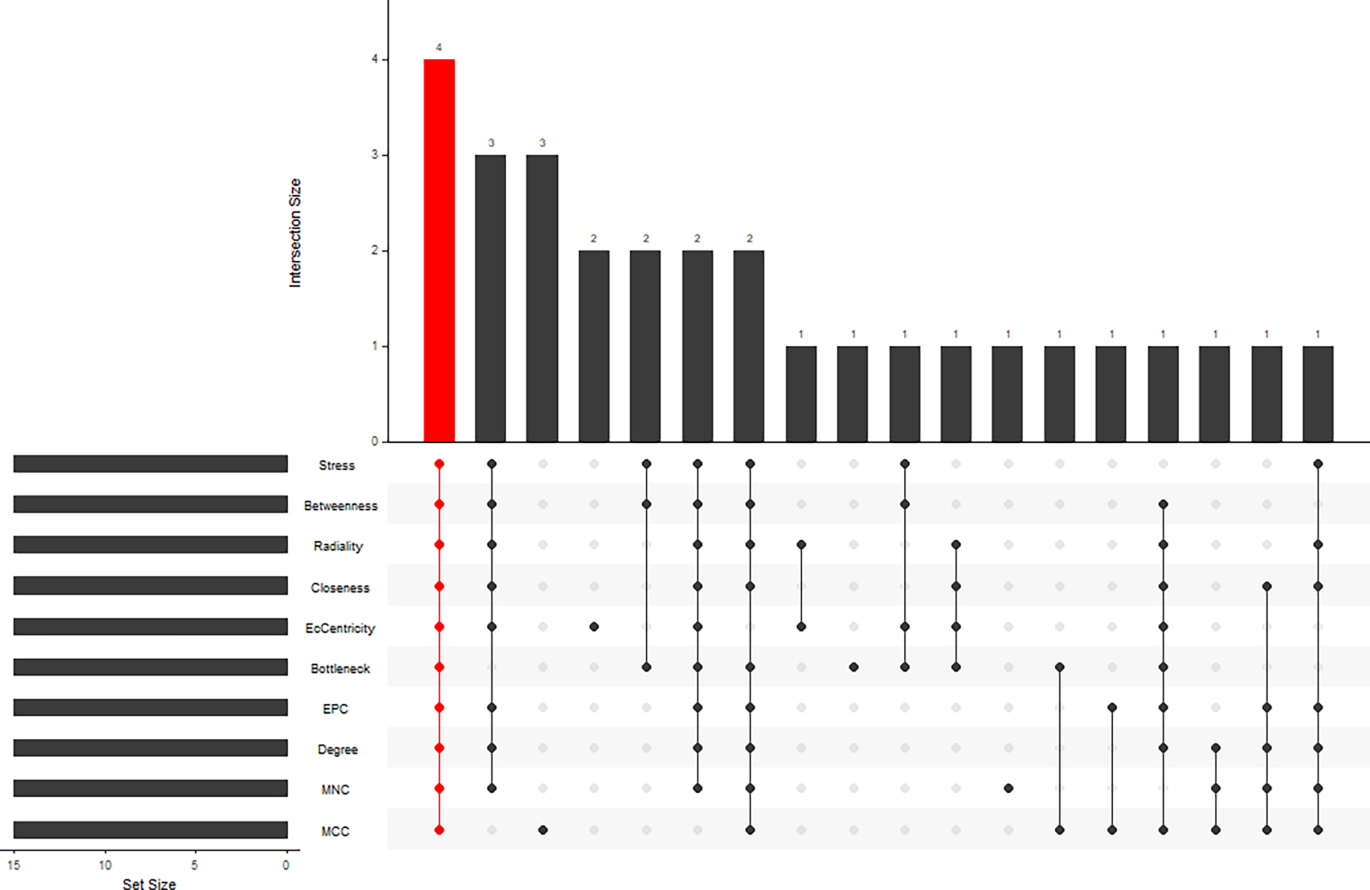
Ten algorithms to screen hub genes by “UpSetR” package.

STRING (https://string-db.org/) online database was used to analyse the 55 integrated DEGs and to construct a PPI network with medium confidence (score > 0.4), as shown in **Figure 4**. The results were downloaded for further analysis by using Cytoscape (v 3.9.0) software. The cytoHubba plugin of Cytoscape was used to score each node gene by 10 randomly selected algorithms, including MCC, MNC, EPC, Degree, BottleNeck, Closeness, EcCentricity, Radiality, Betweenness, and Stress. The top 15 hub genes from each algorithm were identified, and then the common hub genes of the 10 algorithms were selected as hub genes by using the “UpSetR” package, as shown in **Figure 5**. Finally, we identified four hub genes (*FN1*, *C1QA*, *C1QB*, and *CD44*) by the “UpSetR” package.

### Screening and Verification of the Biomarker

**Figure 6:**
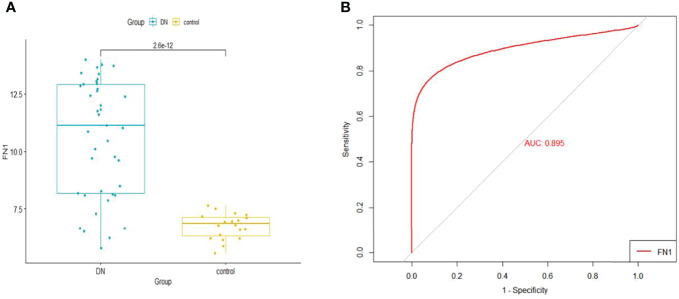
**(A)** The expression of *FN1* in GSE96804. **(B)** ROC curve of *FN1* in GSE96804. ROC, receiver operating characteristic.

When logistic regression analysis was performed on the four genes in the Merge dataset, *FN1* demonstrated a statistically significant difference (p < 0.05); therefore, *FN1* was identified as a biomarker and treatment target. In order to make the results more reliable, we used the dataset GSE96804 for validation. As shown in **Figure 6A**, *FN1* showed a significantly higher expression in DN.

### Receiver Operating Characteristic Curve Analysis

The GSE96804 dataset was used to validate the diagnostic effectiveness of *FN1* for DN by ROC analysis. The larger the area under the ROC curve (AUC), the more the capability of the biomarker to diagnose DN with excellent specificity and sensitivity. As shown in **Figure 6B**, the AUC value of FN1 was 0.895.

### Correlation Analysis and Enrichment Analysis

**Figure 7:**
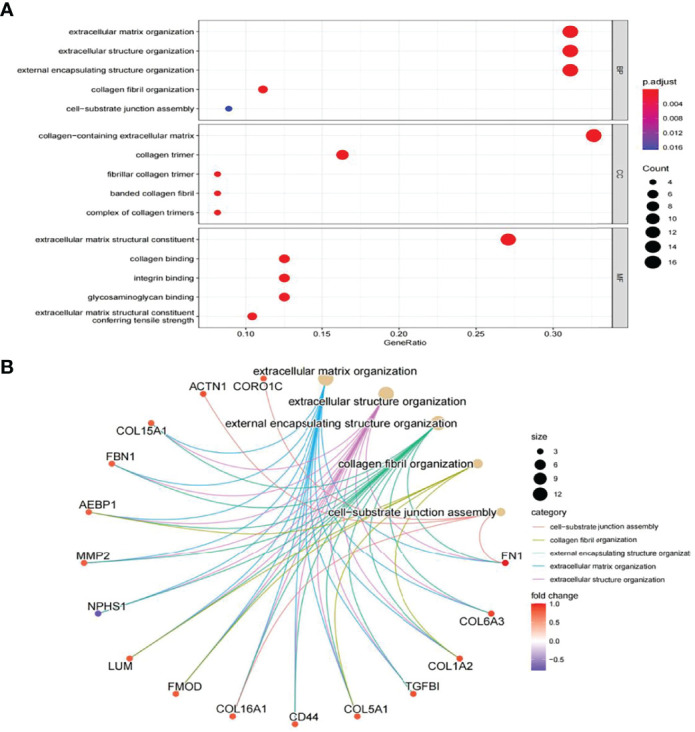
GO enrichment result of top 50 genes. **(A)** The results of GO were presented by bar plot. The x‐axis represents gene ratio, and y‐axis represents GO terms. The size of circle represents gene count. Different colours of circles represent different adjusted p-values. **(B)** The results of GO are presented by circle charts. Different colours of circles represent different correlation coefficients. GO, Gene Ontology.

**Figure 8:**
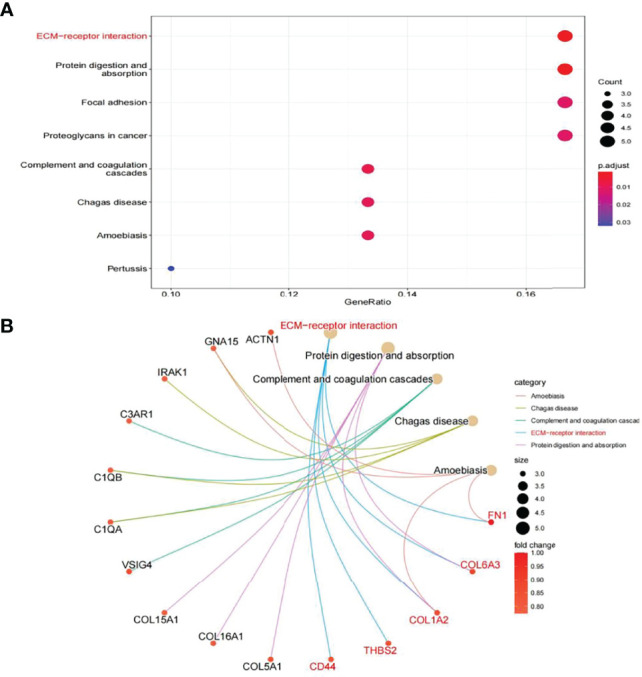
KEGG enrichment result of top 50 genes. **(A)** The results of KEGG were presented by bar plot. **(B)** The circle charts present the results of KEGG. Different colours of the circle represent different correlation coefficients.

**Figure 9:**
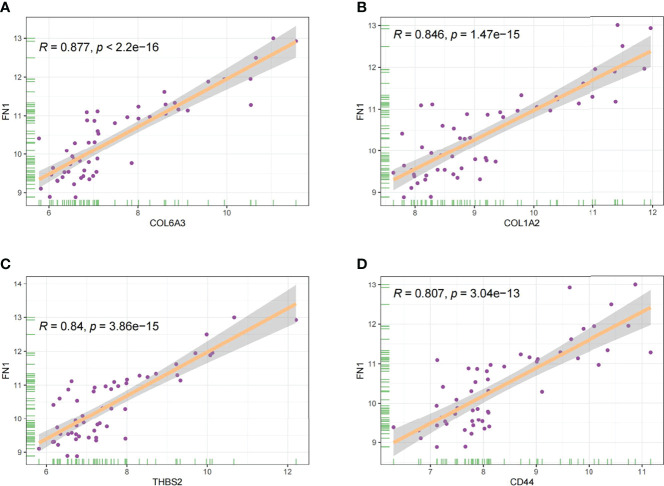
The correlation between *FN1* and *COL6A3* **(A)**, *COL1A2* **(B)**, *THBS2* **(C)**, and *CD44* **(D)**.

**Figure 10:**
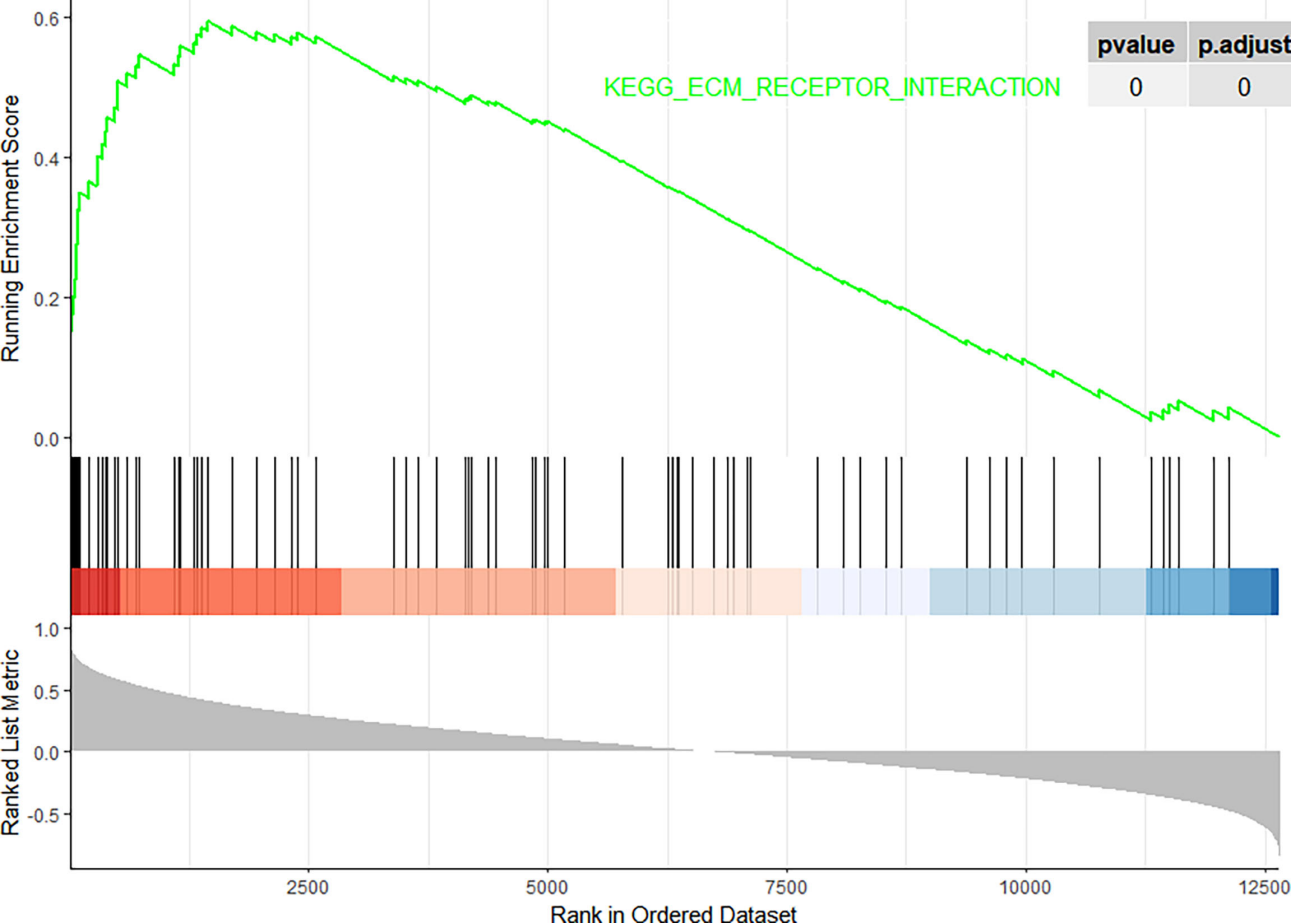
Gene Set Enrichment Analysis (GSEA). The pathway related to *FN1*.

In this study, the correlation between *FN1* and all other genes was analysed by Pearson’s correlation analysis, and the top 50 genes with the highest absolute correlation coefficients were selected for GO and KEGG enrichment analyses. GO functional analysis of the top 50 genes was divided into the following three parts: biological process (BP), molecular function (MF), and cellular component (CC). KEGG is a widely used database for gene research, which links genomic information with higher-order functional information to identify the significantly enriched biological pathways (10). In our study, GO functional annotation and KEGG pathway enrichment analysis were performed using the clusterProfiler package, and P.adjust < 0.05 was considered statistically significant. The results of GO and KEGG are shown in **Figures 7**, **8**, respectively. GO functional enrichment analysis showed that the top 50 genes were mainly involved in the extracellular matrix (ECM) organisation, extracellular structure organisation, external encapsulating structure organisation, collagen-containing ECM, and ECM structural constituent. KEGG pathway analysis revealed that the top 50 genes were significantly enriched in ECM–receptor interaction. Circle charts demonstrated that the five genes were significantly enriched in ECM–receptor interaction, and the four genes other than *FN1* were significantly positively correlated with *FN1*, as shown in **Figures 9A** **–D**. GSEA also showed that *FN1* was significantly enriched in ECM–receptor interaction as shown in **Figures 10**.

## Discussion

DN is the leading cause of end‐stage renal disease globally (11). Due to the extremely complex metabolic disorders that occur in patients with DN, once DN has reached the terminal stage, it is often more difficult to treat than other kidney diseases. Although many studies have investigated the pathogenesis of DN, it has not been clarified completely (12). Therefore, it is necessary to identify potential biomarkers for early diagnosis and targeted therapy of DN. This study used multiple microarrays to conduct bioinformatics analysis for identifying target genes and pathways involved in the occurrence and development of DN. The results of this study suggest that *FN1* may play a key role in the pathogenesis of DN.

In our study, 55 overlapping DEGs were identified between renal glomerular tissues of DN patients and normal controls based on three microarray datasets. Through PPI network analysis and ten algorithms, four hub genes (all upregulated) were finally screened out. The results of logistic regression analysis revealed that *FN1* had a significant relationship with DN (p < 0.05). ROC curve analysis showed that *FN1* had a good predictive ability for DN, suggesting that *FN1* plays an important role in the development of DN. To further understand the functions of *FN1*, we inferred its functions based on the genes that were the most closely associated with its expression, which is called “guilt of association.” The top 50 genes with the greatest absolute correlation coefficients in correlation analysis were selected for GO and KEGG enrichment analyses.

GO and KEGG enrichment analyses were used to explore the molecular mechanisms of the top 50 genes involved in the occurrence and development of DN as well as to determine the potential role of *FN1* in the occurrence and development of DN. GO annotation result of the top 50 genes showed that ECM organisation, extracellular structure organisation, external encapsulating structure organisation, collagen fibril organisation, and cell−substrate junction assembly were chiefly enriched in BP, while CC included collagen-containing ECM, collagen trimer, fibrillar collagen trimer, banded collagen fibril, and complex of collagen trimers. It partly clarifies the intricacy of the pathogenesis of DN and that it destroys several cell components (13). In addition, MF of the top 50 genes mainly involved ECM structural constituent, collagen binding, integrin binding, glycosaminoglycan binding, and ECM structural constituent conferring tensile strength. The above results suggest that *FN1* may be involved in these MFs and influence the progression of DN.

On performing KEGG enrichment analysis, we found that the top 50 genes were mainly involved in ECM–receptor interaction, protein digestion and absorption, focal adhesion, proteoglycans of cancer, complement and coagulation cascades, Chagas disease, amoebiasis, and pertussis. As the most significantly enriched pathway, circle charts showed that five genes (*FN1*, *COL6A3*, *COL1A2*, *THBS2*, and *CD44*) were enriched in ECM–receptor interaction. In addition, *FN1* was significantly positively correlated with *COL6A3*, *COL1A2*, *THBS2*, and *CD44.* According to “guilt of association,” we can infer that the abnormal expression of these genes may be jointly involved in the pathogenesis of DN. GSEA also showed that *FN1* plays a key role in ECM–receptor interaction. Therefore, we can infer that the co-expression of *COL6A3*, *COL1A2*, *THBS2*, *CD44*, and *FN1* plays a key role in the ECM–receptor interaction pathway and promotes the progression of DN.

The ECM is made up of functional macromolecules and a complex mixture of structural components. It is well known that the ECM signalling pathway plays an important role not only in the morphogenesis of tissues and organs but also in the maintenance of cell and tissue structures as well as their functions (14, 15). ECM is also involved in growth, development, and wound repair, and its dynamic components can arbitrate a range of signals, in addition to regulating cell migration and proliferation (16). As an ECM protein-encoding gene, thrombospondin-2 (*THBS2*), encodes a secreted ECM glycoprotein, which can reflect the severity of tissue fibrosis (17, 18).

*COL1A2* is a member of the type I collagen genes and encodes the proα2(I) chains. It has been reported that the upregulation of *COL1A2* in diabetic kidney disease (DKD) can lead to increased renal fibrosis (19). *COL6A3*, a canonical collagen VI gene, encodes the collagen VI isoform. Collagen VI beaded microfibrils have been discovered in the ECM of almost all tissues (20, 21). Collagen VI is highly expressed in a range of cancers and promotes tumour growth and progression. It also influences the tumour microenvironment by augmenting the recruitment of macrophages and endothelial cells, thus enabling tumour inflammation and angiogenesis (22). However, its role in DN is not well understood. CD44 is a well-known cell-surface glycoprotein that is involved in diverse biological pathways, such as cell migration, proliferation, and lymphocyte activation (23, 24). Research has shown that CD44 plays an important role in the pathogenesis of experimental crescentic glomerulonephritis and collapsing focal segmental glomerulosclerosis (25). From PPI network analysis, we found that CD44 was associated with COL1A2, COL6A3, and FN1. This suggests that they all play a key role in the progression of DN.

*FN1* encodes fibronectin, and there are two forms of fibronectin, soluble and insoluble, such as a glycoprotein present in a soluble dimeric form in the plasma, and dimeric or multimeric form at the cell surface and in the ECM. The encoded preproprotein is proteolytically processed to generate the mature protein. Fibronectin is related to cell adhesion and migration processes including embryogenesis, wound healing, blood coagulation, host defence, and metastasis (26). Some studies have shown that overexpression of *FN1* promotes fracture healing by activating the TGF-β/PI3K/Akt signalling pathway (27). In a study on pre-eclampsia, it was found that excess FN1 can promote apoptosis and autophagy in vascular endothelial cells (28). Fibronectin is one of the important components of the ECM. Studies have revealed that FN1 plays a key role in glomerular sclerosis and fibrosis in chronic kidney disease (29, 30). The α5β1 integrin receptor and FN dimer combine to form an FN matrix, which can induce conformational changes in FN and promote the interaction of FN–FN to form new fibrils (31). Through continuous FN deposition, these fibrils grow into a stable insoluble matrix, and based on this, other ECM components are deposited (32). Therefore, the overexpression of *FN1* plays a key role in the progression of fibrosis. Under mechanical stress, *FN1* is upregulated in podocytes (33). Thus, we speculate that *FN1* may play a key role in the disease progression of DN. Evidence from previous studies indicates that *FN1* may be related to the development of DN and thus has the potential to be used as a diagnostic marker of DN. However, clinical studies are needed to verify the diagnostic value of *FN1.*

## Conclusion

In summary, we investigated the key gene for DN using integrated bioinformatics analysis. We identified four candidate genes: *FN1*, *C1QA*, *C1QB*, and *CD44*. On further investigating the biological functions of *FN1*, we found that *FN1* was positively correlated with *THBS2*, *COL1A2*, *COL6A3*, and CD44 and was involved in the development of DN by acting on the ECM receptor interaction pathway. *THBS2*, *COL1A2*, *COL6A3*, and *CD44* are expected to be new research targets for DN, which may provide new directions for the diagnosis and treatment of DN. Consequently, further studies on these genes are warranted. Our findings can contribute to a better understanding of the pathogenesis of DN as well as the development of new therapeutic targets for DN. However, there are several limitations of our study. First, hub genes and pathways were identified based on small sample sizes by bioinformatics analysis. Second, our study did not include experiments to verify the expression of the identified hub genes in DN. All in all, the identified genes and related pathways can increase our understanding of the occurrence and development of DN. However, the biological functions and molecular mechanisms of the genes require further study.

## Data Availability Statement

The datasets presented in this study can be found in online repositories. The names of the repository/repositories and accession number(s) can be found in the article/supplementary material.

## Author Contributions

(I) Conception and design: ZL and YZ. (II) Administrative support: YZ ,XG and JF. (III) Collection and assembly of data: ZL, JF, and ML. (IV) Data analysis and interpretation: ZL. (V) Manuscript writing: all authors. (VI) Final approval of manuscript: all authors.

## Funding

This study was funded by the National Natural Science Foundation of China (81800675), Guangzhou medical key subject construction project of China (2021-2023) and The Program of Huadu District Science and Technology (No.20-HDWS-019).

## Conflict of Interest

The authors declare that the research was conducted in the absence of any commercial or financial relationships that could be construed as a potential conflict of interest.

## Publisher’s Note

All claims expressed in this article are solely those of the authors and do not necessarily represent those of their affiliated organizations, or those of the publisher, the editors and the reviewers. Any product that may be evaluated in this article, or claim that may be made by its manufacturer, is not guaranteed or endorsed by the publisher.
